# Acetylcholine Esterase Inhibitory Effect, Antimicrobial, Antioxidant, Metabolomic Profiling, and an In Silico Study of Non-Polar Extract of The Halotolerant Marine Fungus *Penicillium chrysogenum* MZ945518

**DOI:** 10.3390/microorganisms11030769

**Published:** 2023-03-16

**Authors:** Heba El-Sayed, Marwa A. Hamada, Ahmed A. Elhenawy, Hana Sonbol, Asmaa Abdelsalam

**Affiliations:** 1 Botany and Microbiology Department, Faculty of Science, Helwan University, Helwan 11795, Egypt; 2 Chemistry Department, Faculty of Science, Al-Azhar University, Nasr City, Cairo 11884, Egypt; 3 Chemistry Department, Faculty of Science and Art, Al Baha University, P.O. Box 1988, Mukhwah, Al Baha 6531, Saudi Arabia; 4 Department of Biology, College of Science, Princess Nourah Bint Abdulrahman University, P.O. Box 84428, Riyadh 11671, Saudi Arabia

**Keywords:** Acetylcholinesterase (ACh) inhibition, antimicrobial activity, 2, 2-diphenyl-1-picryl-hydrazyl-hydrate (DPPH) assay, gas chromatography-mass spectrometry (GC/MS), in silico study, *Penicillium chrysogenum* MZ945518, halotolerant

## Abstract

Major health issues, such as the rise in oxidative stress, incidences of Alzheimer’s disease, and infections caused by antibiotic-resistant microbes, have prompted researchers to look for new therapeutics. Microbial extracts are still a good source of novel compounds for biotechnological use. The objective of the current work was to investigate marine fungal bioactive compounds with potential antibacterial, antioxidant, and acetylcholinesterase inhibitory effects. *Penicillium chrysogenum* strain MZ945518 was isolated from the Mediterranean Sea in Egypt. The fungus was halotolerant with a salt tolerance index of 1.3. The mycelial extract showed antifungal properties against *Fusarium solani* with an inhibitory percentage of 77.5 ± 0.3, followed by *Rhizoctonia solani* and *Fusarium oxysporum* with percentages of 52 ± 0.0 and 40 ± 0.5, respectively. The extract also showed antibacterial activity against both Gram-negative and Gram-positive bacterial strains using the agar diffusion technique. The fungal extract was significantly more effective with *Proteus mirabilis* ATCC 29906 and *Micrococcus luteus* ATCC 9341; inhibition zones recorded 20 and 12 mm, respectively, compared with the antibiotic gentamycin, which recorded 12 and 10 mm, respectively. The antioxidant activity of the fungus extract revealed that it successfully scavenged DPPH free radicals and recorded an IC_50_ of 542.5 µg/mL. Additionally, it was capable of reducing Fe^3+^ to Fe^2+^ and exhibiting chelating ability in the metal ion-chelating test. The fungal extract was identified as a crucial inhibitor of acetylcholinesterase with an inhibition percentage of 63% and an IC_50_ value of 60.87 µg/mL. Using gas chromatography–mass spectrometry (GC/MS), 20 metabolites were detected. The most prevalent ones were (Z)-18-octadec-9-enolide and 1,2-Benzenedicarboxylic acid, with ratios of 36.28 and 26.73%, respectively. An in silico study using molecular docking demonstrated interactions between the major metabolites and the target proteins, including: DNA Gyrase, glutathione S-transferase, and Acetylcholinesterase, confirming the extract’s antimicrobial and antioxidant activity. *Penicillium chrysogenum* MZ945518, a halotolerant strain, has promising bioactive compounds with antibacterial, antioxidant, and acetylcholinesterase inhibitory activities

## 1. Introduction

Antibiotic resistance has become a problem for our society and public health because it has made it possible for infectious diseases to come back and pose a threat to people’s health [1]. Many chronic conditions, including cancer, diabetes, arteriosclerosis, neurological illnesses, and heart illnesses, are believed to result from the oxidative damage that free radicals inflict [2]. Therefore, finding secondary metabolites having biological effects against cancer, microbes, tropical diseases, and other conditions has been the focus of extensive research [3].

Marine microorganisms are a possible sustainable source of novel physiologically active compounds because the biodiversity of the oceans makes up 50% of the total biodiversity of the world [4]. Marine microbes are a source of intriguing secondary metabolites because they thrive in challenging environments, including cold, dark, and high pressures, or in conjunction with other species [5]. To survive in such diverse environments, they have developed a variety of adaptation methods, including the development of specific metabolites [4]. Furthermore, marine organisms are able to produce a broad variety of novel molecules due to the sea’s harsh chemical and physical circumstances; these molecules are unique in diversity, structural properties, and functional aspects compared to compounds isolated from terrestrial plants [6]. These extra molecules are a source of possible new pharmaceutically active drugs [7]. *Cladosporium, Aspergillus*, *Chaetomium*, *Penicillium*, and *Trichoderma* species possess a combination of morphological and physiological adaptations that make them well suited to life in the sea. This group of organisms is classified as “facultative marine fungi” [8]. The most prevalent fungi found in both indoor and outdoor habitats, including marine substrates, such as sponges, corals, algae, and sand, are *Penicillium* species [9]. *Penicillium* species that are derived from marine habitats are possible sources of distinctive substances with biological activity that are generated as a result of the natural circumstances of marine environments [8]. There are numerous species within the genus, some of which are commercially important in nutrition, biomedical, and pharmaceutical production [10]. Due to the abundance of bioactive components, such as flavonoids, alkaloids, minerals, proteins, phenols, tannins, vitamins, and antioxidant characteristics, these organisms were able to biosynthesize a diverse array of primary and secondary metabolites [11]. Anticancer, antibacterial, and antioxidant effects are demonstrated by many species of this genus [12]. 

*Penicillium chrysogenum* is a well-known and excellent example of useful fungi. The fungus piqued biologists’ interests, especially in the realm of drug development, because it produces the antibiotic penicillin [13]. The genetic variations among and within the varieties of the species have been studied. James Scott [14] categorized the indoor isolates from *Penicillium chrysogenum* into four clades, and Bank et al. [15] investigated the genetic variance between different types and isolates from *P. chrysogenum.* The metabolites isolated from different varieties of the species showed antioxidant, antimicrobial, and anticancer activities [10,16]. 

The current study set out to investigate the gas chromatography–mass spectrometry (GC/MS) -based metabolic profiling, and the antimicrobial, antioxidant, and acetylcholinesterase inhibition activities of the non-polar ethyl acetate extract of *Penicillium chrysogenum MZ945518* mycelia isolated from the Mediterranean Sea with a docking study of the major bioactive metabolites.

## 2. Materials and Methods

### 2.1. The Fungal Culture Used

*Penicillium chrysogenum MZ945518* was isolated from the Mediterranean coast of Alexandria, Egypt and identified using molecular techniques, as we previously described [17]. After 7 days of cultivation on potato dextrose agar medium, morphological characteristics were examined, and the developed colony was examined under light microscope.

### 2.2. Halotolerance Test

Frisvad’s modified procedure was used to conduct the halotolerance test [18]. Potato dextrose agar (PDA) was employed as the growth medium in this study and supplemented with 0, 2.5, 5, 10, 15, 20, 25, and 30% NaCl concentrations. The fungus was seeded in the plate center and cultured at 28 °C for 10 days, after which the growth diameter was measured. The salt tolerance index (Ti) was determined by dividing the diameters of colonies grown in PDA and colonies grown in PDA plus NaCl. Ti values were found to be oppositely related to halophily. This means that the more halophily there is, the lower the Ti value. Fungi with index values less than one were deemed halophilic, whereas those with index values greater than one were deemed halotolerant.

### 2.3. Extraction of Fungal Metabolites

The *P. chrysogenum* discs (6 mm) were placed in flasks with potato dextrose broth and cultured for 7 days at 25 °C in a static incubator. Thereafter, mycelia were harvested by ultracentrifugation (Sigma, 3–16 PK, Osterode am Harz, Germany) for 10 min at 4 °C and 10,000 rpm, and the culture supernatant was discarded. The obtained mycelia were extracted with ethyl acetate (Sigma-Aldrich, Burlington, MA, United States) solvent (EtOAc) (1:2). At 40 °C, the resulting extract was concentrated using a rotary evaporator (IKA, Germany).

### 2.4. The Antimicrobial Effect Evaluation

#### 2.4.1. The Reference Pathogens

The antibacterial effect of the fungal ethyl acetate extract was evaluated in vitro against six different reference bacterial strains belonging to both Gram-negative and Gram-positive (*Pseudomonas aeruginosa* ATCC 7853, *Proteus mirabilis* ATCC 29906, *Escherichia coli* ATCC 25922, *Staphylococcus aureus* ATCC 25923, *Streptococcus pneumoniae* ATCC 49619, and *Micrococcus luteus* ATCC 9341). Additionally, the extract’s anticandidal properties have been evaluated against the pathogenic yeast *Candida albicans* ATCC 20231. The extract’s antifungal activity was evaluated using three different phytopathogenic fungi (*Rhizoctonia solani*, *Fusarium oxysporum*, and *Fusarium solani*). The tested fungus was maintained on PDA medium at 25 °C for 3–5 days.

#### 2.4.2. Agar–Diffusion Technique

According to Hamad et al. [19], the antibacterial and anticandidal activities of the crude extract were evaluated as follows: 100 μL of the previously cultured bacterial and Candida albicans suspensions, each containing 1 × 10^8^ CFU/mL (OD_600_~0.1) were distributed onto the surfaces of nutrient agar and PDA media, respectively. A 6-mm sterile cork borer was used to produce wells in the agar plates. By using an independent sterile micropipette, 100 µL of the non-polar mycelium extract at a concentration of 20 mg/mL was placed into each well. Then, the plates were kept in the refrigerator at 4 °C for 8 h, followed by incubation at 37 °C for 24 h. Both antibacterial (gentamycin at 10 g/disc) and antifungal (amphotericin B at 100 units/disc) drugs, as well as ethyl acetate were utilized as positive and negative controls, respectively. A ruler was used to measure the diameter of the inhibition zones formed around the wells to determine the antimicrobial efficacy.

#### 2.4.3. Screening of Antifungal Effect

The antifungal activity was determined based on the inhibitory percentage effect on radial mycelial growth (PIMG) of the fungi under investigation, according to Naglah et al. [20], as follows: At first, before pouring the plates, the ethyl acetate extract at a concentration of 20 mg/mL was added to the cooled potato dextrose agar medium (PDA). Then, the media was poured into the plates and left to solidify, and the centers of the plates were then inoculated with fungus discs measuring 5 mm in diameter. A negative control was created by inoculating sterile PDA medium with agar plugs of the same diameter from the investigated fungi. At 25 °C, all cultures were grown for 7 days. Radius of mycelium growth on PDA medium supplemented with mycelium crude extract (R2) was compared to that of mycelium growth on PDA medium (R1) to determine the efficacy of the antifungal properties of the extract. The PIMG was calculated by the formula below:

PIMG = {(R1 − R2)/R1} × 100.

### 2.5. Antioxidant Activity

Numerous procedures were employed in order to evaluate the extract’s antioxidant capacity.

#### 2.5.1. Measurement of Free Radical Scavenging Activity

The 2, 2-diphenyl-1-picryl-hydrazyl-hydrate (DPPH) free radical test was performed as follows by Boly et al. [21]: 100 µL of newly made DPPH reagent was combined with 100 µL of different concentrations of the fungal extract (800, 600, 400, 200, and 100 µg/mL in ethanol); for each concentration, six replicates have been performed. For 30 min, the experiment was conducted at room temperature in the dark. At 540 nm, we observed a decrease in DPPH color intensity. As a positive standard, trolox was dissolved in methanol and prepared at 50, 40, 30, 20, 15, 10, and 5 μM concentrations. According to the following formula, data has been measured as means ± SD:

$$\%\;of\;inhibition=\frac{\left(\mathrm{Average}\;\mathrm{absorbance}\;\mathrm{of}\;\mathrm{blank}-\mathrm{average}\;\mathrm{absorbance}\;\mathrm{of}\;\mathrm{the}\;\mathrm{sample}\right)}{\mathrm{Average}\;\mathrm{absorbance}\;\mathrm{of}\;\mathrm{blank}}\times 100$$

The data was recorded using a FluoStar Omega microplate reader. Microsoft Excel^®^ was utilized in the process of data analysis. Half-maximal inhibitory concentration (IC_50_) was calculated using GraphPad Prism 6^®^ by first logarithmizing the concentrations and then selecting the non-linear inhibitor regression equation (log (inhibitor) vs. normalized response—variable slope equation).

#### 2.5.2. Ferric Reducing Antioxidant Power (FRAP) Assay

With slight adjustments, the FRAP assay was performed in accordance with [22]. The 2,4,6-Tris(2-pyridyl)-s-triazine (TPTZ) reagent was initially freshly prepared using the following ingredients: (300 mM acetate buffer (PH = 3.6), 10 mM TPTZ in 40 mM HCl, and 20 mM FeCl3, in a ratio of 10:1:1 *v*/*v*/*v*, respectively). Then, the reaction was performed in 96 wells plate by mixing 190 µL of the reagent with 10 µL of the sample (at a concentration of 2 mg/mL in methanol), and the reaction was kept at room temperature for 30 min in the dark. The obtained blue color was measured at 593 nm. The data are shown as means ± SD. A 1 mM stock solution of trolox in methanol was used as a positive control. Next, seven serial dilutions were made, decreasing the initial concentration from 800 µM to 600 µM, then 400 µM, 200 µM, 100 µM, and finally 25 µM. 

#### 2.5.3. Metal Ion Chelating Activity

The metal ion chelating test of the fungal non-polar extract has been performed in accordance with the procedure described by [23], with a few minor adjustments made. Briefly, 20 µL of the freshly made ferrous sulphate (0.3 mM) was combined with 50 µL of the fungal extract (1 mg/mL in methanol) in a 96-well plate (with six replicates). Following that, 30 µL of ferrozine at a concentration of 0.8 mM was supplemented to each well. The reaction mixture was ready to measure the change in color intensity at a wavelength of 562 nm after 10 min of room temperature incubation. Using a stock solution of 0.1 mM EDTA in water, five serial dilutions were carried out, resulting in final concentrations of 5, 10, 20, 30, 40, and 50 µM. According to the following equation, data are shown as means ± SD:

$$\%\;of\;inhibition=\frac{\left(\mathrm{Average}\;\mathrm{absorbance}\;\mathrm{of}\;\mathrm{blank}-\mathrm{average}\;\mathrm{absorbance}\;\mathrm{of}\;\mathrm{the}\;\mathrm{sample}\right)}{\mathrm{Average}\;\mathrm{absorbance}\;\mathrm{of}\;\mathrm{blank}}\times 100$$

### 2.6. Acetylcholine Esterase Inhibitory Effect

The acetylcholine esterase (AChE) inhibitory effect was performed with only a few adjustments to the method described by [24] as the following steps: Following the addition of 10 μL of an indicator solution containing 0.4 mM in buffer (1): 100 mM tris buffer pH = 7.5, 20 μL of an enzyme solution containing acetylcholine esterase enzyme (Sigma Aldrich, Inc. St. Louis, MO, USA), 0.02 U/mL in buffer (2): 50 mM tris buffer pH = 7.5 with 0.1% bovine serum albumin were added. The sample solution was then mixed with 140 µL of buffer (1), yielding final concentrations of 0.1 mg/mL and 0.01 mg/mL, respectively. The mixture was allowed to incubate for fifteen minutes at room temperature. After that, 10 μL of the substrate (0.4 mM acetylcholine iodide in buffer 1) was immediately added. The mixture was kept at room temperature in darkness for a period of 20 min. Once incubation was complete, the color was measured at 412 nm. The sample attained an inhibition percentage greater than fifty percent, was subjected to additional testing to establish an IC_50_ value and was prepared with the following final concentrations: 100, 50, 25, 10, and 5 μg/mL. Donepezil was used as a positive standard in methanol at concentrations ranging from 1.0 to 7.0 μg/mL. The data are shown with a mean and a standard deviation.

### 2.7. Chemical Analysis

#### 2.7.1. Determination of Total Phenolics and Flavinoids

The quantity of phenolic metabolites in the ethyl acetate extract of the fungus was determined by using Folin reagent Ciocalteu’s method, described by [25]. Briefly, a mixture of 2.5 mL of Ciocalteu’s Folin reagent, 2 mL of Na_2_CO_3_ (7.5%), and 0.5 mL of fungal extract was prepared and incubated at 25 °C for fifteen minutes. The sample’s absorbance was measured at 765 nm. The total phenolic content was evaluated in terms of milligrams of gallic acid equivalent (GAE) per gram of dry extract using the gallic acid standard curve. Total flavonoid amount was determined using a method described in [26]. Briefly, 0.1 mL of a 10% aluminum chloride solution and 0.1 mL solution of 1 M potassium hydroxide were added to 2 mL of methanol that contained 0.1 mg/mL of a fungal extract. The absorbance of the mixture was measured at 415 nm after it had been incubated at 25 °C for 30 min. Quercetin equivalents (QE) were used to quantify the flavonoids found; the results were calculated in milligrams of quercetin per gram of dry extract.

#### 2.7.2. Gas chromatography–Mass Spectrometry (GC–MS) Analysis

A TRACE GC Ultra Gas Chromatograph (Thermal Scientific Corp., USA) was employed for GC–MS analysis. It was connected to an ISQ Single Quadrupole Mass Spectrometer and a TR-5 MS column (30 m × 0.32 mm i.d., 0.25 m film thick-ness). Helium was used as the carrier gas with a flow rate of 1.0 mL/min and a split ratio of 1:10. The temperature was set to 60 °C for 1 min, then to 240 °C at a rate of 4.0 °C/min per minute for 1 min. The injector and the detector were held at 210 °C. In the injection, 1 µL of the mixtures were diluted (1:10 hexane, *v*/*v*). Mass spectra with m/z ranges of 40–450 were determined using electron ionization (EI) at 70 eV. Metabolites were identified using AMDIS software (www.amdis.net, accessed on 20 December 2022), which relied on retention indices (relative to n-alkanes C8-C22), mass spectra corresponding to authentic standards (when available), the Wiley spectral library collection, and the NSIT library database (accessed on 20 December 2022).

### 2.8. Molecular Modelling

#### 2.8.1. Small Molecule Preparation

The 3D-structures of compounds were optimized using the PM3 (RHF spin state) semi-empirical Hamiltonian molecular orbital computation MO-PAC16 software, which was used in the MOE.2015 package [27].

#### 2.8.2. Protein Structure Selection

In order to fix the active site problems brought on by the structure preparation procedure in MOE, docking experiments were performed using MOE 2015. After the adjustment, hydrogens were added, and the partial charges (Amber12: EHT) were estimated. The energy was minimized (AMBER12: EHT, root-mean-square gradient: 0.100) for targeting proteins including: DNA Gyrase (PDB; 6M1J), glutathione S-transferase (13GS), and Acetylcholinesterase (PDB ID: 1ACJ).

#### 2.8.3. Analysis of Binding Sites

The binding site for the receptor was found using the MOE Site Finder program, which uses a geometric technique to determine potential binding sites in a protein based on its tridimensional structure. Instead of using energy models, this method makes use of alpha spheres, a generalization of convex hulls. The predictions of the MOE Site Finder module were in agreement with the binding sites defined by the co-crystallized ligands in the holo forms of the proteins under investigation.

#### 2.8.4. The Stepwise Docking Method of MOE

The enzymes’ crystal structure was determined. They applied an MMFF94x force field to the parameters and charges. The triangular matcher placement method, which generates poses by aligning ligand triplets of atoms on triplets of alpha spheres represented in the receptor site points, was applied to the optimized 3D structure of the molecule. During each iteration, a random triplet of alpha sphere center was used to determine the pose. The position created was once more assessed using the London dG. approach. Using the MMFF94x force field, the poses were improved, and solvation effects were taken into account. The Born solvation model (GB/VI) was used to calculate the final energy, and the free energy in Kcal/mol was used to assign a grade to each final position.

#### 2.8.5. ADMET Profile

Swiss ADME (http://www.swissadme.ch/, accessed on 6 January 2023) provided the ADMET (absorption, distribution, metabolism, elimination, and toxicity) profile for compounds. The Lipinski rule of five (Molecular weight, logarithms of partial coefficient, hydrogen bond donor (HBD), and hydrogen bond acceptor (HBA)) was used to first screen the profiled compounds for their physicochemical properties to find the Pharmaceutical Active Ingredients (PAIs). From PubChem (https://pub-chem.ncbi.nlm.nih.gov, accessed on 6 January 2023), the canonical SMILES for the molecular structures of each of the metabolites were retrieved. Pharmacokinetic properties were further selected out of the compounds with desirable physicochemical characteristics.

### 2.9. Statistical Analysis

Every test was conducted three times, with each run including three independent replicates. The data were subjected to analysis of variance (ANOVA), and group averages were compared using Fisher’s exact test (*p* ≤ 0.05). The software Minitab^®^ was utilized to carry out the statistical analysis.

## 3. Results

### 3.1. Morphological Macroscopic and Microscopic Characters of the Isolated Fungus

**Figure 1:**
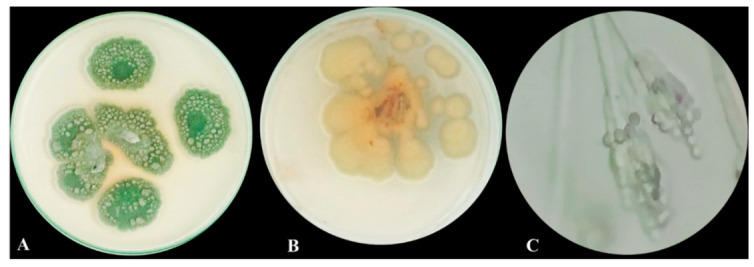
Morphological and microscopic examination of *P*. *chrysogenum* MZ945518. (**A**) Upper view of the fungal colony, (**B**) Dorsal view of fungal colony, and (**C**) Fungal conidiophore under light microscope.

After seven days of colony development at 25 °C on PDA (potato dextrose agar) medium, colonies were 30–45 mm in diameter, had heavy sporulation, were mostly deep green in the middle and surrounded by a white border with an irregular edge, and the back was mostly a pale yellowish color and clear exudate droplets were observed (Figure 1A,B). A branched conidiophore with chains of conidia was observed under a light microscope (Figure 1C).

### 3.2. Halotolerance Test

**Figure 2:**
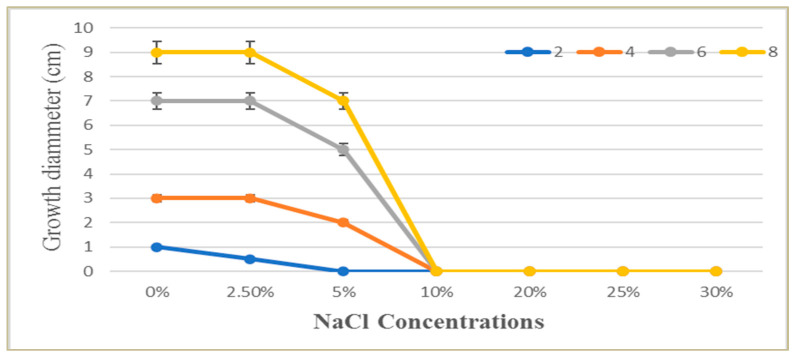
The effect of different NaCl solution concentrations (0.0–30%) on the growth of *P. chrysogenum* MZ945518 at different incubation times (2, 4, 6 and 8 days).

Based on its salt tolerance levels, *P*. *chrysogenum* MZ945518 was classified as halotolerant or halophilic using the salt tolerance index (Ti). *P*. *chrysogenum* was grown on PDA and PDA supplemented with 2.5, 5, 10, 20, 25 and 30% NaCl plates. Ti value was 1.3 at NaCL 5%, which indicated the studied fungus was halotolerant. The fungal growth on the PDA plates with 2.5% NaCl after two, four, and six days of incubation were similar to the fungal growth of the control plates (Figure 2). Growth diameter was reduced by 5 and 10% NaCl and completely inhibited by higher concentrations. 

### 3.3. Antimicrobial Activity

*P. chrysogenum* MZ945518 ethyl acetate extract was studied for its antimicrobial effect against six bacterial reference strains, including Gram-negative and Gram-positive bacteria, as well as one yeast strain. The diameter of the fungal extract’s inhibitory zone (mm) against the tested bacteria and yeast was compared to that of the commercial antibiotics, gentamycin and amphotericin B, as shown in Table 1. The fungal extract presented significantly higher activity against the two bacterial strains, *Micrococcus luteus* ATCC 9341 and *Proteus mirabilis* ATCC 29,906, by producing zones with diameters of 20 and 12 mm, respectively, compared with the antibiotic gentamycin. Moreover, the extract killed *Streptococcus pneumoniae* ATCC49619 in a way that was almost the same as gentamycin. Additionally, the extract demonstrated efficacy against the remaining investigated bacteria and the yeast; however, its effects were moderate or negligible in comparison to those of gentamycin and amphotericin B.

For fungi, PIMG was evaluated to test the antifungal activity of ethyl acetate extract versus three plant pathogenic fungal species, and the results showed that the mycelial growth of *Fusarium solani* was the most affected by the *P. chrysogenum* MZ945518 extract with an inhibitory percentage of 77.5 ± 0.3. (Table 2).

### 3.4. Antioxidant Activity

The antioxidant capacity of the ethyl acetate extract of the studied fungus was measured with the DPPH-free radical scavenging method, the ferric reducing antioxidant power (FRAP) assay, and metal ion chelating activity. The findings of DPPH scavenger activity on fungal ethyl acetate extracts revealed that the IC50 value against DPPH radicals was 542.5 ± 69.1 μg/mL (Table 3). Moreover, the results of the FRAP test demonstrated that the fungal extract was converted from Fe^3+^ to Fe^2+^, although the values were less impressive than those obtained with the Trolox compound. Moreover, the results of the metal ion chelating activity revealed that the extract had a lower chelating ability when compared to conventional EDTA solutions with 12.7 ± 0.9 μM EDTA eq/mg extract.

### 3.5. Acetylcholine Esterase Inhibitory Effect

The effectiveness of the fungus extract in inhibiting the acetylcholinesterase enzyme (AChE) exhibited a clear suppression of enzyme activity (63% inhibition percentage) and a recorded IC_50_ value of 60.87 3.8 µg/mL (Table 4).

### 3.6. Chemical Analysis

#### 3.6.1. Total Phenolics and Flavonoids

Using the Folin–Ciocalteau and aluminum chloride techniques, respectively, the total phenolic and flavonoid content in the ethyl acetate extract of the *P. chrysogenum* MZ945518 was determined. Flavonoids and phenolics were present in totals of 133.4 and 373.5 mg/g, respectively.

#### 3.6.2. GC/MS Profiling

**Figure 3:**
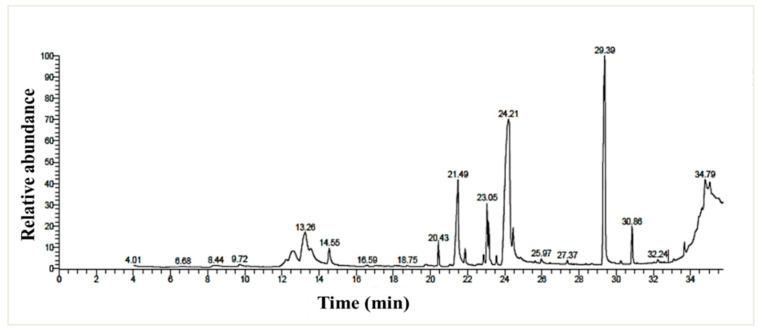
GC/MS chromatogram of the *P. chrysogenum* MZ945518 non-polar ethyl acetate extract.

The chemical profiling of the *P. chrysogenum* MZ945518 extract was performed using a GC/MS instrument (Figure 3). By matching the retention time and mass spectra to either authentic data standards or data from the Wiley spectral library and the NSIT library database, twenty metabolites were detected in the ethyl acetate extract (Table 5). With a ratio of 36.28%, (Z)-18-octadec-9-enolide was the most abundant metabolite, followed by 1, 2- Benzenedicarboxylic acid with a ratio of 26.73%. n- Hexadecanoic acid (7.8%), 2, 3-dihydroxypropyl acetate (5.3%), 9, 12-octadecadienoic acid (Z, Z)-, methyl ester (4.8%), and butyl 9, 12, and 15-octadecatrienoate (3.2%) were also major metabolites.

### 3.7. Molecular Docking Study

**Figure 4:**
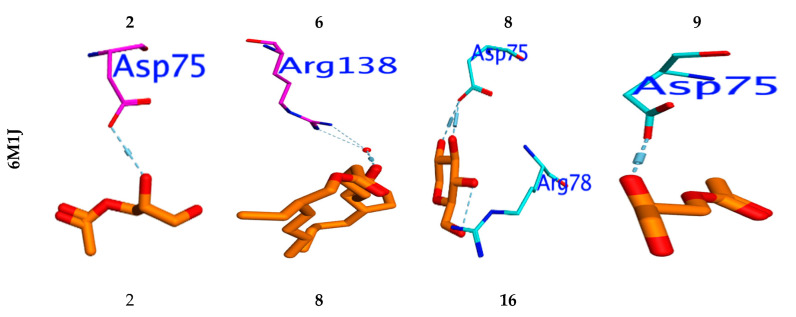

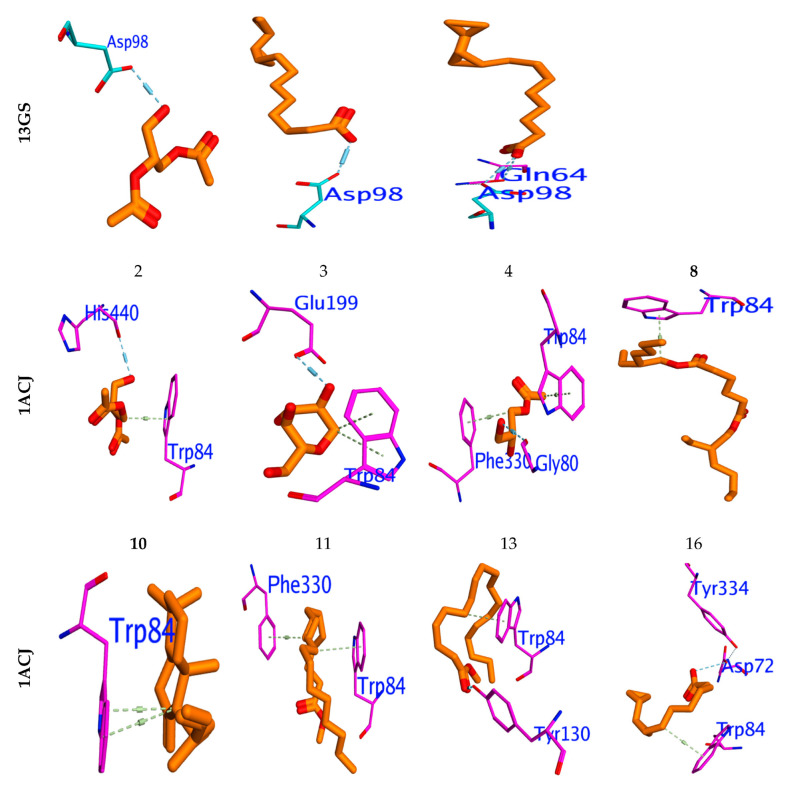
Binding mode for metabolites with the highest expected sharing in antibacterial (6M1J), antioxidant (13GS), and acetylcholinesterase “AChE” (PDB ID: 1ACJ activity.

To investigate the in silico antimicrobial inhibition action of isolated compounds from fungus ethyl acetate extract, the docking study was applied against DNA Gyrase proteins. The different docking energies were listed in (Table 6). The metabolites two, six and eight showed the highest binding energy in (Kcal/mol.), as they were −7.78, −7.33, and −7.76 against the DNA Gyrase (PDB; 6M1J; [28]). The isolated compounds 3–5, 7, and 9–20 showed moderate binding efficiency against 6M1J enzymes. All compounds stabilized in the active binding site (ASP75, ARG78 & ARG138) in a similar way to the reference inhibitor (Figure 4).

Molecular docking was performed to examine which isolated compounds displayed antioxidant activity. Compounds two, eight, and sixteen exhibited the highest binding energy against 13GA (−6.19, −6.51, and −6.13 Kcal/mol, respectively). These compounds stabilized in the binding pocket by forming a strong H-bond with the essential amino acid Asp98 (Figure 4). All isolated compounds occupied the binding pocket (ASP98, GLN64, LEU52, ARG13, SER65, PRO53) with the same type of reference inhibitor. Furthermore, the molecular docking performed against Acetylcholinesterase’s active site, “AChE” (PDB ID: 1ACJ), post-docking results showed that all docked identified molecules revealed a binding efficacy ΔG in the range of −4.95 to −8.72 Kcal/mol. The isolated pa2, 3-dihydroxypropyl acetate (5) has the highest binding efficacy (ΔG = −8.72 kcal/mol) among all isolated components, while the glyceryl acetate (1) showed the lowest binding efficiency (−5.82 kcal/mol). The validity of the docking experiment was confirmed by the low RMSD value (0.86 to 1.92), as represented in Table 6.

### 3.8. ADMET Profile

The pharmacological and pharmacokinetic features of the molecule must reach the action point in a timely manner, in an adequate concentration, and be able to be cleared from the body after their action 17. As a result, the in silico ADME properties of the compounds are crucial in drug discovery. Using the Swiss ADME profile, in silico ADME computational investigations were carried out. The hydrogen bond acceptor/donor (HBA/HBD), solubility, lipophilicity, topological surface area (TPSA), and percentage of absorption (%ABS) of all the drug-like properties have been identified. The %ABS was achieved by the following formula: %ABS = 109 – (0.345 × TPSA). Table 7 illustrates the data that were attained. Lipinski’s rule of five states that molecules with the following characteristics—hydrogen bond donors fewer than five and hydrogen bond acceptors fewer than ten—can have greater in vivo absorption and bioavailability. These criteria include molecular weight below 500 and estimated log P less than five. Substances that break more than one of the aforementioned rules may have bioavailability issues. According to the computational ADME results, all of the detected compounds showed Log P values between 3.20 and 4.05, which indicates good cell permeability. With the exception of compounds 18 and 19 (MW = 728 and 537), all of the compounds have molecular weights under 500, indicating simple delivery and absorption.

## 4. Discussion

Microorganisms that can live and thrive in harsh environments are thought to be a bountiful origin of various naturally occurring bioactive and novel molecules. One of the challenging environmental factors that microorganisms must adapt to in order to thrive is high salt. Adaptation involves the overproduction of bioactive metabolites and, at times, the synthesis of novel biochemicals which can be utilized as new antioxidants and antimicrobial sources [29]. This study relied on *P. chrysogenum* MZ945518 isolated from the Mediterranean Sea in Egypt. The fungus was identified genetically in a previous study [17]. In our findings, additional morphological studies were conducted on the fungus, and the results revealed that in the front view it had heavy sporulation, was mostly deep green in the middle, and was surrounded by a white border with an irregular edge. In the back view, it was mostly a pale yellowish color, and clear exudate droplets were observed on PDA plates. Additionally, under a light microscope, it showed a branched conidiophore with chains of conidia. Previous studies [30] reported that on PDA media, *Penicillium chrysogenum* displayed modest development, with the colony’s backside being colored yellow and its core being green. Finding the halotolerance index of *P. chrysogenum* MZ945518, which was isolated from the Egyptian Mediterranean shore, was one of the goals of this study. The results of the test revealed that the growth pattern of the fungus and calculations of the medium tolerance index demonstrated that the fungus is thought to be permanently halophilic. Genus *Penicillium* compressed many members, which are classified as extremophiles [31]. Various strains of the fungus *P. chrysogenum* have reportedly been isolated and survive in excessively salty habitats [32,33].

In general, many *Penicillium* species produce various chemical types of secondary metabolites, some of which are significant in the field of medicine, others of which are used to produce mycotoxins, significant drugs, and some of which are used in industry, particularly in the production of penicillin [32,33]. Our findings involved studying the different activities (the antimicrobial, antioxidant, and acetylcholine esterase inhibitory effect) of the *Penicillium chrysogenum* MZ945518 crude extract. According to a report published by the World Health Organization in 2022, antimicrobial resistance is one of the top ten global public health concerns facing humanity (https://amrcountryprogress.org/#/visualization-view, accessed on 6 January 2023). Many antimicrobial drugs have lost their potency in recent years. This highlights the critical need for further research into novel antimicrobial sources and metabolites. The fungal non-polar extract showed antibacterial action against all of the pathogenic organisms that were tested, whereas, in comparison to the commercial antibiotic gentamycin, the activity was most powerful against *Micrococcus luteus* and *Proteus mirabilis*. *Proteus mirabilis* is considered a human pathogen which infects the urinary tract, especially in people who have long-term hospitalization [34,35]. Previous research [36] found that *P. chrysogenum* has the superior antibacterial activity against nine bacterial species, including *Escherichia coli*, *Acinetobacter baumannii*, and *Staphylococcus aureus*, when compared to *Aspergillus oryzae* and *Aspergillus niger*. Furthermore, the fungus inhibited *Pseudomonas aeruginosa* growth significantly [37]. Additionally, [38] reported that on cheap mediums, such as grape waste and cheese whey, the strain *P. chrysogenum* IFL1 developed active metabolites with antibacterial, antifungal, and amoebicidal properties. The metabolite xanthocillin isolated from *P. chrysogenum* demonstrated strong inhibitory activities against *Klebsiella pneumoniae*, *Acinetobacter baumannii*, and *Pseudomonas aeruginosa* [39]. Moreover, a compound called citrinin, which is made from the fungus *Penicillium chrysogenum* FF001 and originally found in the sponge *Melophlus* sp., is effective against drug-resistant strains of *Staphylococcus aureus* and *Enterococcus faecium* [40]. Our results suggest that the *P. chrysogenum* extract can be used to combat the human pathogen *Candida albicans*. Several human diseases, such as cancer and inflammatory disorders, have been related to *C. albicans* [41]. According to research that was published in [37], *P. chrysogenum* makes a protein that can stop *C. albicans* from growing. Al-Saleem et al. [42] showed that *Penicillium chrysogenum* extract was highly effective in killing both *Candida albicans* and *Staphylococcus aureus*. Halotolerance fungi are known to have a biologically active substance which possesses antimicrobial activities [29].

Antifungal activity of the fungal ethyl acetate extract showed that the fungal possesses antifungal bioactivity against *Fusarium oxysporum, Rhizoctonia solani,* and *Fusarium solani.* Both plants and animals are susceptible to infection by these pathogenic fungi. *Fusarium oxysporum* is one of the most harmful plant pathogens around; it can also infect humans and is increasingly being recognized as a major health threat because of its capacity to cause severe illness in those with impaired immune systems [43]. *Rhizoctonia solani* is a devastating fungus that attacks economically significant crops all around the world [44]. *Fusarium solani* causes rot in a wide variety of crops, including citrus, rice, peas, beans and potatos. Pathogenic fungi can be combated with biocontrol strategies rather than fungicide, which is less harmful to ecosystems [45,46,47]. The antifungal activity of the *Penicillium chrysogenum* protein has been stated by [48,49]. *P. chrysogenum* IFL1 metabolites could inhibit *Fusarium* spp. and other phytopathogenic fungi [12]. Biological control of *Fusarium oxysporum* using *Bacillus velezensis* [50] and *Streptomyces sp.* [51] has been reported. *Streptomyces and Bacillus spp* was found to be effective for biological control of *Rhizoctonia solani* [52,53].

In the current investigation, a non-polar extract from *P. chrysogenum* showed antioxidant activity based on the DPPH free radicals, FRAP test and metal ion chelating activity. Antioxidants play an important role in cell protection by blocking free radicals at their active site and trapping free radicals that cause degenerative processes [54,55]. Previous research has demonstrated that *Penicillium chrysogenum* has a strong antioxidant capacity [11,42]. The antioxidant activity of *P. chrysogenum* MZ945518 has an IC_50_ of 542.5 µg/mL in the DPPH test. According to results from DPPH in [56], the whole extract of *P. chrysogenum* has an antioxidant activity IC_50_ of 1086.2 µg/mL. 

The GC/MS analyzer was used to determine the fungus’s chemical profile, which resulted in the identification of twenty metabolites, the majority of which have biological functions. For example, metabolites biformen has anti-inflammatory activity [57]. Palmitic acid showed potent inhibitory activity against both Gram-positive and Gram-negative bacteria [58,59]. Methyl palmitate has a nematocidal effect, antifibrotic, and anti-inflammatory activities [60,61,62]. 9,12-Octadecadienoic acid (Z, Z)-, methyl ester has analgesic, anti-inflammatory, and ulcerogenic properties [63]. 11-Octadecenoic acid, methyl ester has antidiarrhoeal activity [64]. Methyl palmitate and methyl stearate have been shown to be nematicidal against *Meloidogyne incognita*, an insect pest of bananas [61]. 1,2- Benzenedicarboxylic acid has antimicrobial activity [65].

The fungal extract in this investigation was capable of inhibiting the acetylcholinesterase enzyme (AChE) at a rate of 63%. Our findings contradict the findings of [66], who reported that after testing fifteen compounds isolated from *Penicillium chrysogenum* for their ability to inhibit AChE, the results revealed that none of them had any effect on the enzyme’s activity. [67] reported that *Penicillium sp.* metabolites significantly decreased acetylcholinesterase activity in *Culex quinquefasciatus* and *Aedes aegypti larvae*, compared to *Aspergillus sp. and Rhizopus sp.* We obtained better results than [56], which said that *Penicillium janthinellum* extract only blocked AChE by 36.62%. Acetylcholine is considered one of the best-studied neurotransmitters and has been linked to Alzheimer’s disease pathogenesis (neurodegenerative disease and the leading cause for dementia), and its hydrolysis is catalyzed by AChE. From this vantage point, blocking the enzyme responsible for producing AChE has proven to be an efficient Alzheimer’s disease treatment [68,69]. Natural products have been shown to have anti-AD efficacy and AChE inhibition in a variety of preclinical and clinical studies [70]. Several sources, including [71], have reported that marine species play a role as a source of AChE inhibitor metabolites. 

A docking study revealed that the compositions of glycerol 1,2-diacetate, 2,3-bis (Acetyloxy)-1-[(acetyloxy)methyl] propylacetate, and palmitic acid are most commonly shared in antimicrobial activity via the inhibition of DNA gyrase and interaction with essential amino acid residues, such as Arg78, Glu79, and Thr167, for DNA gyrase. The assumption that the inhibitory efficiency for the studied compositions increased with increasing hydrophilicity resulted from the variance in the interaction mode between compositions and hydrophilic amino acid backbones. In addition, glycerol 1, 2-diacetate, palmitic acid, [1, 1’-Bicyclopropyl]-2-octanoic acid, 2’-hexyl, and methyl ester are among the most identified antioxidant substances. According to its crystallographic structure, the AChE (PDB [72] has two major binding sites: the catalytic active site (CAS) and the gorge-connected peripheral anionic site (PAS) [73]. The PAS is composed of (Tyr70, Asp72, Tyr121, Trp279, and Phe290), whereas the CAS is composed of (Ser200, Glu327, and His440), the anionic substrate (Trp84, Glu199, and Phe330), and the acyl binding pocket (Phe288 and Phe299) [72]. All substances interacted with the essential amino acid residue Trp84 at the binding site in the same way as the reference inhibitor. When applied to the AChE domain, the notable substances two, three, four, eight, ten, eleven, thirteen, and sixteen caused biological inhibition potency. These substances have the highest binding activities. According to the Swiss ADME’s (http://www.swissadme.ch/, accessed on 6 January 2023) ADMET results, all hybrids except for seven and eighteen and nineteen conform to the Lipinski requirements because the number of hydrogen bond acceptors and donors present in the hybrids was fewer than ten. Compounds 1–5, 7–17, and 19–20 should be easily absorbed by the human body, with the exception of compounds 6 and 18. All conjugates, with the exception of six and eighteen, had percentage absorption values more than 74%. These findings show that, with the exception of 7–18, compounds 3–12 have favorable pharmacokinetic characteristics and minimal toxicity.

## 5. Conclusions

Mycelium extract from the halotolerant *Penicillium chrysogenum* strain MZ945518 was antimicrobial potent. It was effective against various strains of Gram-positive and Gram-negative bacteria, with *Proteus mirabilis* ATCC 29,906 and *Micrococcus luteus* ATCC 9341 exhibiting the strongest activity. Moreover, it demonstrated antifungal characteristics against different pathogenic fungi. The DPPH, FRAP, and MIC assays have all revealed that the fungal extract had potent antioxidant activity. Additionally, it was shown to have an inhibiting effect on acetylcholine esterase. The non-polar extract comprises a variety of bioactive molecules with several biological activities, according to the results of the GC–MS study of fungal metabolites. The docking and ADME studies showed favorable pharmacokinetic characteristics and minimal toxicity. These remarkable results provide a path to more investigation into using these compounds in drug preparation and searching for other natural bioactive compounds obtained from marine fungi.

## Figures and Tables

**Table 1:** The antibacterial and anticandidal effect of ethyl acetate extract of *P. chrysogenum* MZ945518 by well diffusion technique.

Microbial Pathogens		Inhibition Clear Zone Diameter (Mm)		
		*P. Chrysogenum* Extract	Gentamycin (10 μg/disc)	Amphotericin B (100 units/disc)
Gram-positive bacteria	*Staphylococcus aureus* ATCC25923	11 ± 0.2	21 ± 0.2	Nt
	*Micrococcus luteus* ATCC 9341	20 ± 0.6 *	18 ± 0.1	Nt
	*Streptococcus pneumoniae* ATCC49619	14 ± 0.8	15 ± 0.5	Nt
Gram-negative bacteria	*Escherichia coli* ATCC25922	20 ± 0.0 #	25 ± 0.8	Nt
	*Pseudomonas aeruginosa* ATCC 7853	13 ± 0.5	20 ± 0.6	Nt
	*Proteus mirabilis* ATCC29906	12 ± 0.2 *	10 ± 0.0	Nt
Pathogenic yeast	*Candida albicans* ATCC 20231	15 ± 0.1	Nt	20 ± 0.5

The results were reported as the mean ±standard deviations of three independent replicates. *: Designates significance in comparison to conventional antibiotics (*p* > 0.05); #: significant within the same group; and “Nt” indicates not tested.

**Table 2:** *P. chysogenum* MZ945518 ethyl acetate extract’ antifungal effect.

Percent of Inhibition of Mycelial Growth (PIMG) %		
*Rhizoctonia solani*	*Fusarium oxysporum*	*Fusarium solani*
52 ± 0.0	40 ± 0.5	77.5 ± 0.3

The data for the PIMG percents are means ± SD of three independent replicas.

**Table 3:** Antioxidant activities of *P. chysogenum* MZ945518 ethyl acetate extract.

	DPPH IC_50_ (μg/mL)	FRAP (μM Trolox eq/mg Extract)	MIC (μM EDTA eq/mg Extract)
Fungus extract	542.5± 69.1	57.9 ± 4.6	12.7 ± 0.9
Trolox	24.4 ± 0.8		

**Table 4:** Inhibitory effect of *P. chysogenum* MZ945518 extract on acetylcholine esterase activity.

	% Inhibition 100 μg/mL	IC_50_ (μg/mL)
fungus extract	63.32 ± 2.88	60.87 ± 3.81
Donepezil		3.4 ± 0.32

**Table 5:** The metabolites identified in the *P. chrysogenum* MZ945518’s ethyl acetate extract.

	Compound Name	Chemical Formula	Molecular Weight (g/mol)	Retention Time (min)	Area %
1	Glyceryl acetate	C_5_H_10_O_4_	134	12.22	0.41
2	Glycerol 1,2-diacetate	C_7_H_12_O_5_	176	12.54	1.20
3	1,5-Anhydroglucitol	C_6_H_12_O_5_	164	12.59	1.50
4	2,3-dihydroxypropyl acetate	C_5_H_10_O_4_	134	13.25	5.32
5	1,2,3-Propanetriol triacetate	C_9_H_14_O_6_	218	13.57	0.66
6	2,3-bis (Acetyloxy)-1-[(acetyloxy)methyl] propylacetate	C_12_H_18_O_8_	290	14.55	1.62
7	Methyl palmitate	C_17_H_34_O_2_	270	20.43	1.83
8	n-Hexadecanoic acid (palmitic acid)	C_16_H_32_O_2_	256	21.49	7.80
9	5,7-dimethoxy-1-Naphthalenol,	C_12_H_12_O_3_	204	21.86	1.19
10	4,4,8a-Trimethyl-7-methylidene-8-[(2E)-3-methylpenta-2,4-dienyl]-2,3,4a,5,6,8-hexahydro-1H-naphthalene (Biformen)	C_20_H_32_	272	22.87	0.88
11	9,12-Octadecadienoic acid (Z, Z)-, methyl ester	C_19_H_34_O_2_	294	23.05	4.81
12	11-Octadecenoic acid, methyl ester	C_19_H_36_O_2_	296	23.15	2.91
13	Methyl stearate	C_19_H_38_O_2_	298	23.56	0.77
14	(Z)-18-Octadec-9-enolide	C_18_H_32_O_2_	280	24.21	36.28
15	Octadecanoic acid	C_18_H_36_O_2_	284	24.46	1.79
16	[1,1’-Bicyclopropyl]-2-octanoic acid, 2’-hexyl-, methyl ester 56687-68-4 DTXSID301016055 2’-Hexyl-1,1’-bicyclopropane-2-octanoic acid methyl ester	C_21_H_38_O_2_	322	25.97	0.46
17	Hexanedioic acid, bis(2-ethylhexyl) ester	C_22_H_42_O_4_	370	27.37	0.34
18	1,2- Benzenedicarboxylic acid	C_24_H_38_O_4_	390	29.39	26.73
19	1-Heptatriacotanol	C_37_H_76_O	536	30.26	0.29
20	Butyl 9,12,15-octadecatrienoate	C_22_H_38_O_2_	444	30.86	3.22

**Table 6:** The Docking energy scores (kcal/mol) for the identified molecules.

	ΔG	rmsd	E.vdw	E.Int	*E._H_._B_*	ΔG	Rmsd	E.vdw	E.Int	*E._H_._B_*	ΔG	rmsd	E.vdw	E.Int	*E._H_._B_*
6M1J						13GS					1ACJ				
1	−4.81	1.47	−7.21	−43.20	−8.70	−4.81	1.46	−9.43	−5.83	2.47	−4.95	1.16	−9.58	−8.59	−9.70
2	−7.78	1.63	38.65	−16.40	−8.11	−7.78	1.16	−6.77	20.65	1.63	−8.34	141	9.33	−13.12	−5.96
3	−5.08	1.29	75.79	−50.89	−12.08	−5.08	1.22	−3.81	94.18	1.29	−8.44	1.87	23.75	−19.11	−8.32
4	−5.14	1.24	−7.81	−51.91	−9.07	−5.14	1.30	−3.06	467.93	2.24	−8.72	1.07	27.24	−9.68	−7.02
5	−6.60	1.62	34.15	−41.51	−9.74	−6.60	0.96	−9.61	−1.21	1.62	−5.05	1.89	−8.36	−10.65	−9.55
6	−7.33	1.33	20.53	−57.93	−9.28	−7.33	1.55	−7.88	17.57	1.33	−5.83	0.86	−36.81	−12.70	−9.18
7	−6.52	1.26	151.64	−56.57	−11.23	−6.52	1.77	−8.01	22.37	1.26	−7.72	1.41	28.78	−15.85	−10.59
8	−7.76	1.43	28.39	−41.65	−9.06	−7.76	1.95	−9.56	148.97	1.43	−8.31	1.86	28.54	−14.61	−8.72
9	−6.13	1.65	−74.75	−47.34	−8.27	−6.13	1.38	−6.13	25.10	1.65	−6.80	1.26	170.25	−17.94	−9.60
10	−4.72	1.76	−4.69	−45.57	−8.96	−4.72	1.38	−7.07	−75.53	2.76	−8.80	1.03	30.61	−12.23	−4.94
11	−4.64	1.42	−5.24	−49.54	−9.79	−4.64	1.21	−8.50	−6.66	1.42	−8.54	1.17	37.63	−12.92	−6.88
12	−4.62	1.42	−8.33	−49.96	−9.07	−4.62	1.66	−7.12	16.17	4.42	−5.84	1.76	−4.40	−15.34	−9.33
13	−4.56	1.10	−7.04	−43.75	−8.71	−4.56	1.30	−3.06	467.93	2.10	−8.28	1.88	36.96	−16.97	−9.04
14	−4.84	1.02	74.63	−57.86	−11.22	−4.84	1.17	−11.10	87.17	2.02	−7.71	1.28	61.95	0.03	−1.18
15	−4.77	1.88	77.57	−51.50	−11.51	−4.77	1.53	−8.91	−7.16	1.88	−5.34	1.90	73.59	−12.84	−9.40
16	−4.70	1.61	73.16	−65.51	−11.22	−4.70	1.82	−7.60	−44.23	2.61	−8.25	1.23	−2.90	−9.71	−5.77
17	−5.14	2.24	−7.81	−51.91	−9.07	−5.14	1.45	−8.58	16.83	2.24	−7.58	1.67	33.96	−19.36	−11.18
18	−5.13	1.92	−6.97	−52.68	−10.11	−5.13	1.86	−7.31	24.00	0.92	−6.77	1.92	155.01	−25.88	−9.63
19	−4.84	1.44	−5.89	−47.97	−8.69	−4.84	1.00	−10.09	152.42	1.44	−7.96	1.67	29.16	−17.06	−9.26
20	−4.79	1.10	−8.82	−47.96	−8.68	−4.79	1.41	−6.41	19.07	1.10	−6.83	0.90	44.70	−22.36	−8.49

**Table 7:** Pharmacokinetics of target compounds’ ADMET predictions.

Compd. No	Lipinski Parameters					nROTB ^e^	TPSA ^f^	ABS% ^g^	BBB ^h^	GI ABS ^i^
	MW ^a^	HBA ^b^	HBD ^c^	LogP ^d^	Violations					
1	134.13	4	2	−3.83	0	4	66.76	85.97	High	No
2	176.17	5	1	−3.6	0	6	72.83	83.87	High	No
3	164.16	5	4	−3.13	0	1	90.15	77.90	Low	No
4	134.13	4	2	−3.83	0	4	66.76	85.97	High	No
5	218.2	6	0	−3.45	0	8	78.9	81.78	High	No
6	708.7	18	0	−2.05	2	32	228.86	30.04	Low	No
7	270.45	2	0	−2.71	1	15	26.3	99.93	High	Yes
8	256.42	2	1	−2.77	1	14	37.3	96.13	High	Yes
9	144.17	1	1	−2.16	0	0	20.23	102.02	High	Yes
10	272.47	0	0	−2.86	1	3	0	109.00	Low	No
11	294.47	2	0	−2.86	1	15	26.3	99.93	Low	No
12	296.49	2	0	−2.82	1	16	26.3	99.93	High	No
13	298.5	2	0	−2.19	1	17	26.3	99.93	High	No
14	280.45	2	0	−3.32	1	0	26.3	99.93	High	Yes
15	284.48	2	1	−2.19	1	16	37.3	96.13	High	No
16	322.53	2	0	−2.57	1	15	26.3	99.93	High	No
17	370.57	4	0	−3.7	1	19	52.6	90.85	High	No
18	728.69	18	10	−2.11	3	18	338.86	7.91	Low	No
19	537	1	1	3.55	2	35	20.23	102.02	Low	No
20	334.54	2	0	−3.05	1	17	26.3	99.93	Low	No

^a^ Molecular weight; ^b^ Hydrogen Bond Acceptor; ^c^ Hydrogen Bond Donor; ^d^ Partition Coefficient; ^e^ Number of rotable bonds; ^f^ Topological Polar Surface Area; ^g^ Absorption %; ^h^ Blood Brain Barrier; ^i^ Gastro-intestinal absorption.

## Data Availability

All data generated or analyzed during this study are included in this article.
